# Subgingival microbial diversity and respiratory decline: A cross‐sectional study

**DOI:** 10.1111/jcpe.13819

**Published:** 2023-04-13

**Authors:** Lewis Winning, Gary Moran, Mary McClory, Ikhlas El Karim, Fionnuala T. Lundy, Christopher C. Patterson, Dermot Linden, Kathy M. Cullen, Frank Kee, Gerard J. Linden

**Affiliations:** ^1^ Dublin Dental University Hospital, Trinity College Dublin Dublin Ireland; ^2^ Centre for Experimental Medicine, School of Medicine, Dentistry and Biomedical Sciences Queen's University Belfast Belfast Northern Ireland; ^3^ Centre for Public Health, School of Medicine, Dentistry and Biomedical Sciences Queen's University Belfast Belfast Northern Ireland; ^4^ Centre for Medical Education, School of Medicine Dentistry and Biomedical Sciences Queen's University Belfast Belfast Northern Ireland

**Keywords:** chronic obstructive pulmonary disease, periodontitis, respiratory function, subgingival microbiome

## Abstract

**Aim:**

To investigate whether there is an association between subgingival microbial diversity and reduced respiratory function.

**Materials and Methods:**

A group of dentate 58–72‐year‐old men in Northern Ireland had a comprehensive periodontal examination including subgingival plaque sampling. DNA was extracted from plaque samples and the V1–V3 regions of the 16S rRNA gene were analysed by high‐throughput sequencing and a microbial diversity index (MDI) was derived. Spirometry measurements were made using a wedge bellows spirometer. The primary outcome variable of interest was the percentage of predicted forced expiratory volume in 1 s (% predicted FEV_1_). Analysis included multiple linear regression with adjustment for various confounders.

**Results:**

Five‐hundred and seven men were included in the analysis. The mean age was 63.6 years (SD = 3.1). Of these, 304 (60.0%) men had no or mild periodontitis, 105 (20.7%) had moderate periodontitis and 98 (19.3%) had severe periodontitis. Multiple linear regression analysis showed that a one unit increase in MDI was associated with a 0.71% loss (95% confidence interval: 0.06%–1.35%; *p* = .03) in % predicted FEV_1_ after adjustment for all confounders.

**Conclusions:**

In this group of dentate men from Northern Ireland, subgingival microbial diversity was associated with reduced respiratory function.

**Clinical Relevance**

*Scientific rationale for study*: Epidemiological studies have reported an association between periodontitis and respiratory decline. This study aimed to investigate whether there was an association between subgingival microbial diversity associated with periodontitis and reduced respiratory function.

*Principal findings*: The study showed that in a group of 58–72‐year‐old Caucasian dentate men in Northern Ireland, subgingival diversity (measured using a microbial diversity index) was independently associated with reduced respiratory function.

*Practical implications*: Dentists should be aware of the potential systemic health implications of patients presenting with periodontitis. Subgingival microbial diversity associated with periodontitis may be a risk indicator for reduced respiratory function.

## 1 BACKGROUND

Chronic respiratory diseases, including chronic obstructive pulmonary disease (COPD), asthma and lung cancer, are among the most common non‐communicable diseases worldwide (Labaki & Han, 2020). Chronic respiratory diseases (together) accounted for 3.9 million deaths in 2017 and were responsible for 1,470 disability‐adjusted life‐years (DALYs) per 100,000 individuals, placing them among the leading cause of morbidity and mortality globally (Soriano et al., 2020). The importance of the lung microbiome in the aetiology and progression of chronic respiratory diseases has recently received increased research focus (Man et al., 2017). Contrary to what was previously thought, the lungs are not sterile (O'Dwyer et al., 2016). Advances in next‐generation DNA sequencing have provided the opportunity to study the lung microbiome in both health and disease. In healthy individuals, the lung microbiome largely reflects commensal communities present in the upper respiratory tract, which enter the lungs via microaspiration (Charlson et al., 2011). 16S rRNA studies have shown that, at a family level, the healthy lung microbiome is typified by the presence of *Firmicutes, Bacteriodetes, Proteobacteria, Fusobacteria* and *Actinobacteria*, while at a species level *Prevotella, Veillonella* and *Streptococcus* are characteristic (Moffatt & Cookson, 2017). However, changes in microbial diversity or abundance in the lung microbiome have been associated with several chronic respiratory diseases including COPD, bronchiectasis and asthma (Budden et al., 2019). A major feature of the lung microbiome in disease of the lower airways is a shift in community composition away from the *Bacteroidetes* phylum (which usually dominates) towards *Gammaproteobacteria*, the class that contains many common lung‐associated gram‐negative pathogens (Huffnagle et al., 2017).

By virtue of their close anatomic proximity, the oropharynx–lung continuum represents a potential gateway for microbial exchange. Sub‐clinical aspiration of oropharyngeal contents is acknowledged to occur universally in humans (Gleeson et al., 1997; Huxley et al., 1978). Studies have demonstrated that in healthy individuals, the microbiome of the lungs more closely resembles that of the oropharynx rather than the nasopharynx or the lower gastrointestinal tract (Bassis et al., 2015; Huffnagle et al., 2017; Morris et al., 2013). The oral microbiome is comprised of up to 1,000 prevalent taxa at the species level (Dewhirst et al., 2010; Radaic & Kapila, 2021). Various niches within the oral cavity give rise to a heterogeneous ecological system, affecting the local microbial community (Li et al., 2022). The complex equilibrium between resident species in the oral cavity is responsible for the maintenance of a healthy state (symbiosis) or a state associated with oral disease (dysbiosis) (Kilian et al., 2016). Therefore, dysbiotic change in the oral microbiome, such as that observed in periodontitis, may serve as a persistent reservoir of potential pathogens to the respiratory system.

A possible link between periodontitis and respiratory disease (COPD) was first suggested in epidemiological analyses of the National Health and Nutrition Examination Survey data (Scannapieco et al., 1998; Scannapieco & Ho, 2001) and data from the Veterans Administration Dental Longitudinal Study (VADLS) (Hayes et al., 1998). Evidence, however, for periodontal bacteria impacting the lung microbiome is predominantly indirect and based mainly on studies linking poor oral and denture hygiene to types of pneumonia (Linden et al., 2013; Scannapieco & Cantos, 2016). The main clinical feature of chronic respiratory diseases such as COPD or asthma is reduced respiratory function (Cukic et al., 2012). An association between periodontitis and reduced respiratory function in the general population has previously been reported (Holtfreter et al., 2013; Winning et al., 2019). However, to our best knowledge, no studies have investigated the potential link between periodontitis‐associated microbial diversity and reduced respiratory function. Therefore, the aim of this study was to investigate whether there is an association between subgingival microbial diversity associated with periodontitis and reduced respiratory function in a group of men from Northern Ireland.

## 2 MATERIALS AND METHODS

### 2.1 Study subjects

Subjects investigated in this study were participants in the PRIME study (Prospective Epidemiological Study of Myocardial Infarction), which is a longitudinal cohort study of cardiovascular disease among men in Northern Ireland (Winning et al., 2021). The current study is a cross‐sectional study of a subset of these participants.

**FIGURE 1:**
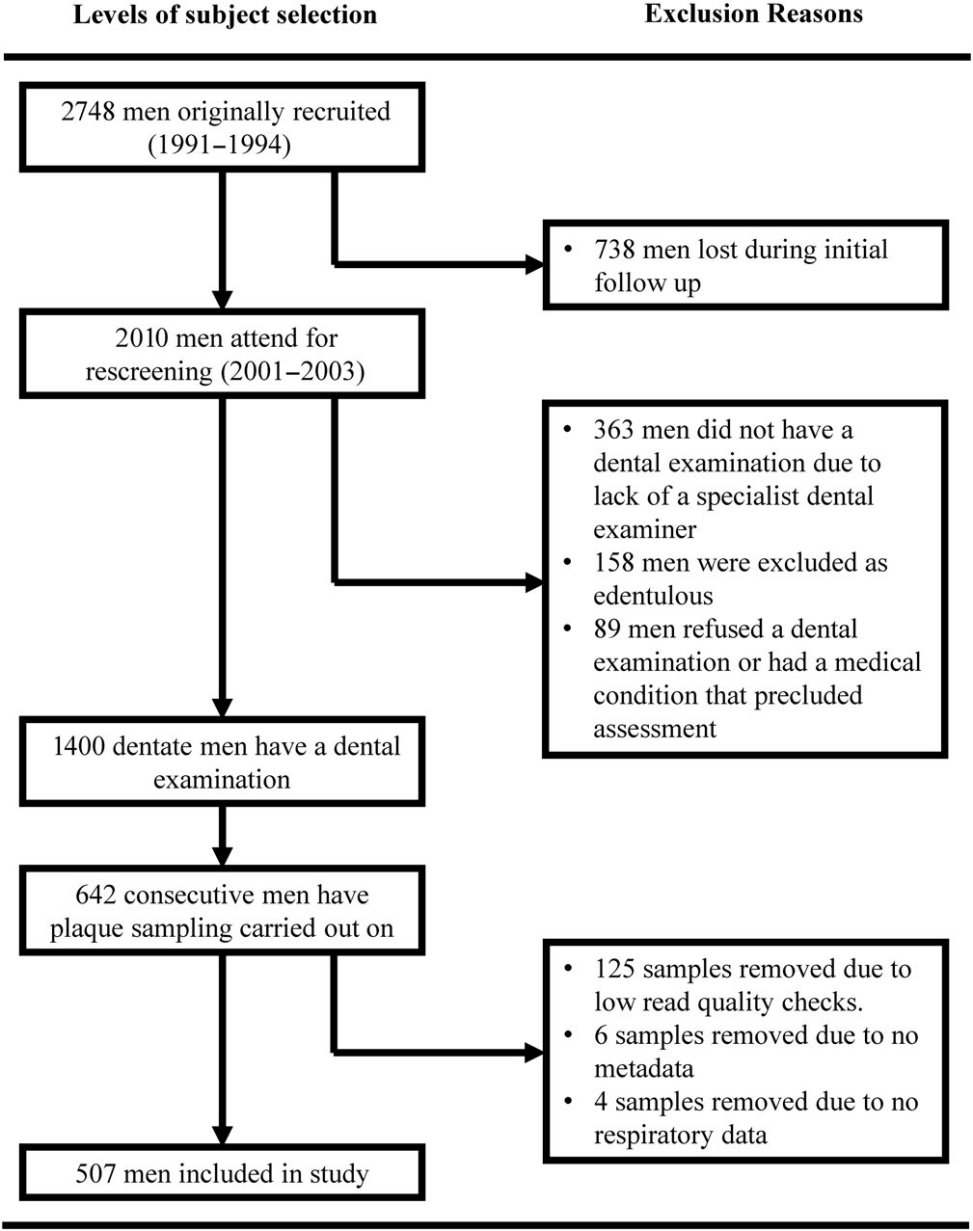
Recruitment and enrolment of study participants.

From 1991 to 1994, 2748 men were recruited from local industry, the civil service and general medical practice. The sample represented ~5% of 50–60‐year‐old men in the Greater Belfast region and broadly matched the social class structure of the population (Yarnell, 1998). Between 2001 and 2003, the surviving men were contacted by post and invited to attend a re‐screening visit as part of their continuing involvement in the PRIME study. A total of 2010 men were reviewed, and a clinical periodontal examination was completed for 1400 (69.7%) of the men. The remainder of the sample was made up of 363 (18.1%) men who did not have a dental examination because a specialist dental examiner was not available during their visit, 158 (7.9%) who were edentulous and 89 (4.4%) who refused or had a medical condition that precluded periodontal probing. Subgingival plaque sampling was carried out on a consecutive sample of 642 men who had a clinical periodontal examination (Winning et al., 2015). Parallel to periodontal examinations, respiratory function was also measured. Analysis in the current study is based on 507 men with complete plaque sequencing data and respiratory data. Men who did not have plaque sampling carried out, had low‐quality plaque sample reads, had missing metadata or missing respiratory data were excluded (Figure 1). A comparison between men included in the analysis and those not is detailed in Tables S1 and S2.

Participants also completed questionnaires gathering information on their medical history, social circumstances, demographic background and tobacco use. A physical examination assessed anthropometric measures including weight, height, waist and hip measurements. Approval for the project was obtained from the Research Ethics Committee of the Faculty of Medicine, Queen's University, Belfast, and the Office for Research Ethics Committees (Northern Ireland). All participants provided informed, written consent. All data used in this study pertain to the examination undertaken during 2001–2003 when each subject attended a periodontal examination, plaque sampling, anthropometric measures, demographic information (questionnaire data) and respiratory function assessments.

### 2.2 Periodontal examinations

All periodontal examinations were completed by one of four dental hygienists who had been calibrated against a ‘gold standard’ set by a senior clinical researcher (GL) prior to the study. Regular monthly meetings took place to ensure inter‐ and intra‐examiner consistency and reproducibility. Throughout the study, the hygienists maintained the standard set at the outset, with κ‐values >0.8 at the regular training sessions (Linden et al., 2009). Clinical periodontal measurements were made using a Michigan O periodontal probe (Hu‐Friedy, Chicago, IL, USA) with Williams markings. Clinical measurements were made at the mesial, distal, buccal and palatal/lingual aspects of all teeth excluding the third molars. Probing pocket depth (PPD) was measured from the gingival margin to the base of the periodontal pocket with the probe tip parallel to the long axis of the tooth. Measurements were made to the nearest millimetre and, when any doubt existed, the lower value was scored. Clinical attachment level (CAL) was recorded as the distance from the cemento–enamel junction (CEJ) to the base of the clinical pocket. This was calculated by measuring the distance from the CEJ to the gingival margin and subtracting this value from the probing depth measurement (recession was recorded as a negative value). Periodontitis status was defined as one of three case definitions: no or mild periodontitis; moderate periodontitis; or severe periodontitis. These case definitions followed the Centers for Disease Control and Prevention and the American Academy of Periodontology (CDC/AAP) classification (Page & Eke, 2007). ‘Severe periodontitis’ required two or more interproximal sites with CAL ≥ 6 mm, not on the same tooth, and one or more interproximal sites with PPD ≥ 5 mm. ‘Moderate periodontitis’ was defined as two or more interproximal sites with CAL ≥ 4 mm, not on the same tooth, or two or more interproximal sites with PPD ≥ 5 mm, not on the same tooth. The ‘No or mild periodontitis’ category included cases that were neither ‘severe periodontitis’ nor ‘moderate periodontitis’.

### 2.3 Subgingival plaque sampling

Three subgingival plaque samples were collected per participant, with sites being chosen based on the deepest PPD (Winning et al., 2015). Where more than three sites had equally deep probing depths, it was left to the hygienist to sample from the most accessible sites. Sites of interest were isolated using cotton rolls, and subgingival plaque samples were obtained using a single stroke of a Gracey curette (Hu‐Friedy) from the base of the pocket. Samples were then placed in 500 μL of sterile phosphate‐buffered saline (PBS) in sterile eppendorfs (Sigma, St. Louis, MO, USA). Samples were immediately transferred for storage at −80°C. DNA was extracted from plaque samples after first being lyophilized, followed by a sequence of enzymatic lysis and addition of Proteinase‐K (Qiagen, Manchester, UK). Following this, total DNA was isolated from the plaque sample using a DNeasy Blood and Tissue kit (Qiagen) according to the manufacturer's instructions. Samples were pooled and processed on a participant (rather than site) basis. The quality and quantity of DNA was determined using a Nanodrop spectrophotometer (Thermo Fischer Scientific, Rockford, IL, USA). All DNA samples were extracted shortly after plaque samples were collected (2001–2003) and were stored at −80°C until required for further analysis.

### 2.4 16S rRNA amplification and sequencing

Sequencing was carried out by the Core Genomic Unit in Queen's University Belfast for all available subgingival plaque DNA samples. For 16S library preparation and sequencing, the V1–V3 region of the 16S rRNA gene was amplified from DNA samples according to the Illumina 16S metagenomics protocol (16S Metagenomic Sequencing Library Preparation, #15044223 B; https://support.illumina.com/documentation.html; Illumina: San Diego, CA, USA). Libraries were sequenced on an Illumina Miseq using a V3 600 Cart, 300bp PE. Amplicon PCR was performed using the primer pair 27F (5′‐AGAGTTTGATCCTGGCTCAG‐3′) and 534R (5′‐ATTACCGCGGCTGCTGG‐3′), each with overhang adapter sequences (IDT, Coralville, Iowa).

### 2.5 Bioinformatics for sequenced data

Bacterial 16S rRNA sequences were processed and filtered using Dada2 (Callahan et al., 2016). Following error estimation and correction using the Dada2 algorithm, paired reads were merged and chimeras were removed following their identification with the ‘removeBimeraDenovo’ command. Taxonomy was assigned using the Human Oral Microbiome Database (HOMD) classifications (eHOMD 16S rRNA RefSeq version 15.1) (Dewhirst et al., 2010). Phyloseq was used to examine the relative abundances of microbial taxa (McMurdie & Holmes, 2013). The DESeq2 package (Love et al., 2014) was used to detect pairwise differences in taxonomic abundances based on periodontitis disease status defined by the CDC/AAP classification (severe periodontitis vs no or mild periodontitis case definitions). Differences were deemed significant if they met two criteria: (i) a log2‐fold difference threshold of 1.2, and (ii) *p*‐value cut‐off of .01 (adjusted for false discovery rate using Benjamini–Hochberg correction), thus limiting it to taxa that differed by at least 30% with a 1% chance of false‐positive identification.

### 2.6 Microbial diversity index

The main exposure variable was the microbial diversity index (MDI). This was generated as the log of (total abundance in organisms increased in severe periodontitis) over (total abundance of organisms decreased in severe periodontitis), based on previous methodology (Gevers et al., 2014). Organisms used for the MDI were the taxa identified as either increased or decreased with periodontitis severity in the DeSeq2 analysis (severe periodontitis vs no or mild periodontitis case definitions).

### 2.7 Spirometry

The research staff involved in spirometry measurement were trained by the respiratory medical staff at the Royal Victoria Hospital, Belfast. Spirometry was performed using a wedge bellows spirometer (Vitalograph S Model, Bucks, UK). The forced expiratory volume in 1 s (FEV_1_) and forced vital capacity (FVC) from the best three attempts were recorded (Winning et al., 2019). Each individual spirometry trace was reviewed for validity using the American Thoracic Society/European Respiratory Society criteria (Miller et al., 2005). The primary outcome variable of interest was the % predicted FEV_1_. This was obtained by comparing the greatest measured FEV_1_ to a reference value, calculated from age, gender, height and race by applying the widely used equation of the European Community for Steel and Coal (Quanjer, 1983).

### 2.8 Definitions of other variables

Anthropometric measurements including body weight (to the nearest 200 g), total waist circumference (to the nearest 0.5 cm) and height (to the nearest cm) were measured by research nurses trained and calibrated according to the PRIME protocol. Participants who reported that they had ever smoked more than 100 cigarettes were questioned about their smoking history. Participants were classified as current, former or never smokers. Hypertension was by self‐report of the condition in response to the question ‘Have you ever been told by a doctor that you have high blood pressure (hypertension)?’ Similarly, diabetes was also by self‐report of the condition. Atherosclerotic cardiovascular disease (ACVD) was recorded for men who had a previous diagnosis of coronary heart disease, ischemic cerebrovascular disease or peripheral arterial disease. Accurate information pertaining to ACVD was available from the main study database (as this is the primary outcome under investigation in the PRIME study, with men having already been under observation for 10 years prior to entry to the current study) (Winning et al., 2020). Education was assessed by the number of years in full‐time education. Socio‐economic conditions were categorized based on three proxy indicators: the type of living accommodation (rented or owned/mortgage), the number of cars/vans/motorcycles in the household and the number of baths and/or showers and toilets in the home. Subjects were categorized as low level of material conditions if the subject was not homeowner and had at most one car, one bath shower and one toilet; high level of material conditions was considered if the subject had at least two cars and was either a homeowner or had two or more baths/showers or two or more toilets; all other people were classified in the intermediate level (Wagner et al., 2003). Toothbrushing frequency was categorized as ‘less than twice per day’ or ‘two or more times per day’.

### 2.9 Statistical analysis

For comparison of baseline characteristics, subjects were divided into thirds by tertiles based on the % predicted FEV_1_ score. Comparison of baseline characteristics were made using either analysis of variance (ANOVA) for continuous data or chi‐square test for categorical variables. The data for mean CAL and mean PPD were not normally distributed, so log‐transformed values were used. For descriptive purposes, these variables were summarized as geometric means (interquartile range) rather than as means (standard deviation), which were used to summarize other continuous variables.

Multiple linear regression was conducted in a sequential model design where potential confounding variables were added to produce a fully adjusted final model incorporating established predictors of % predicted FEV_1_. Model 1 included adjustment for age, waist circumference and smoking; Model 2 included additional adjustment for diabetes, hypertension and ACVD; and, finally, Model 3 included adjustment for socio‐economic conditions, education years and toothbrushing frequency.

The level of statistical significance was set at *p* < .05. Analyses were performed using SPSS version 27 (IBM Corp., Armonk, NY, USA), Stata release 17 (Stata Corp., College Station, SA), and R (R Core Team, Vienna, Austria).

## 3 RESULTS

A total of 507 men were included in the analysis (Figure 1). The mean age of the men was 63.6 years (SD = 3.1), with a range of 58–72 years. Applying the Page and Eke (2007) case definitions of periodontitis, 304 (60.0%) of the men had no or mild periodontitis, 105 (20.7%) had moderate periodontitis and 98 (19.3%) had severe periodontitis. In terms of respiratory function, 98 (19.3%) men had a % predicted FEV_1_ of 50%–80% indicating mild to moderate airflow limitation, while 10 (2%) men had a % predicted FEV_1_ less than 50% indicating more severe airflow limitation. Tertiles divided the % predicted FEV_1_ score into thirds of *n* = 169 men (ranges: 25.5%–85.4%; 85.5%–100.5%; 100.6%–147.7%).

Characteristics of the men by thirds of the % predicted FEV_1_ are reported in Table 1. A strong association across smoking categories was observed (*p* < .001). Across increasing thirds of the % predicted FEV_1_, the proportion of never smokers increased (30.8% vs. 41.4% vs. 48.5%), while the proportion of current smokers decreased (25.4% vs. 10.1% vs. 12.4%). Mean total waist circumference was significantly different across the thirds, with men with a higher % predicted FEV_1_ having a smaller mean waist circumference (*p* < .001). Men with a higher % predicted FEV_1_ were also less likely to have a history of ACVD (*p* = .03). Socio‐economic measures assessed by years in full‐time education were on average higher in men, with a high % predicted FEV_1_ (*p* < .001). Men in the lower third of % predicted FEV_1_ had significantly fewer mean number of teeth (*p* < .001), proportionally more moderate‐to‐severe periodontitis (*p* = .02) and a greater mean CAL (*p* < .001) compared to those in the higher thirds.

**TABLE 1:** Characteristics of cohort studied by the % predicted FEV_1_ thirds (*n* = 507).

% predicted FEV_1_ thirds	<85.5% (*n* = 169)	85.5%–100.5% (*n* = 169)	>100.5% (*n* = 169)	*p*
Age, years, mean (SD)	63.7 (3.1)	63.5 (3.1)	63.5 (3.0)	.75
Smoking, *n* (%)				
Never	52 (30.8%)	70 (41.4%)	82 (48.5%)	**<.01**
Former	74 (43.8%)	82 (48.5%)	66 (39.1%)	
Current	43 (25.4%)	17 (10.1%)	21 (12.4%)	
Total waist circumference (cm), mean (SD)	97.3 (10.9)	96.9 (8.8)	92.4 (8.7)	**<.01**
Diabetes, *n* (%)	10 (5.9%)	15 (8.9%)	9 (5.3%)	.38
Hypertension, *n* (%)	62 (36.7%)	58 (34.3%)	44 (26.0%)	.09
ACVD, *n* (%)	28 (16.6%)	21 (12.4%)	12 (7.1%)	**.03**
Education, years, mean (SD)	11.2 (2.6)	11.9 (3.4)	12.6 (3.4)	**<.01**
Material conditions, *n* (%)				
Low	59 (34.9%)	53 (31.5%)	41 (24.3%)	.16
Medium	44 (26.0%)	36 (21.4%)	47 (27.8%)	
High	66 (39.1%)	79 (47.0%)	81 (47.9%)	
Toothbrushing frequency, *n* (%)				
< 2 times /day	11 (6.5%)	6 (3.6%)	6 (3.6%)	.32
Number of teeth, mean (SD)	18.1 (6.3)	19.4 (5.8)	20.7 (5.3)	**<.01**
Moderate/severe periodontitis, *n* (%)	82 (48.5%)	59 (34.9%)	62 (36.7%)	**.02**
PPD, geometric mean (IQR)	2.2 (1.9–2.6)	2.1 (1.8–2.4)	2.1 (1.8–2.4)	0.09
CAL, geometric mean (IQR)	2.4 (1.7–3.2)	2.1 (1.5–2.9)	1.9 (1.4–2.4)	**<0.01**

*Note*: Bold values signify statistical significance *p* < 0.05.

Abbreviations: ACVD, atherosclerotic cardiovascular disease; CAL, clinical attachment loss; IQR, interquartile range; *n*, number; PPD, probing pocket depth; SD, standard deviation.

**FIGURE 2:**
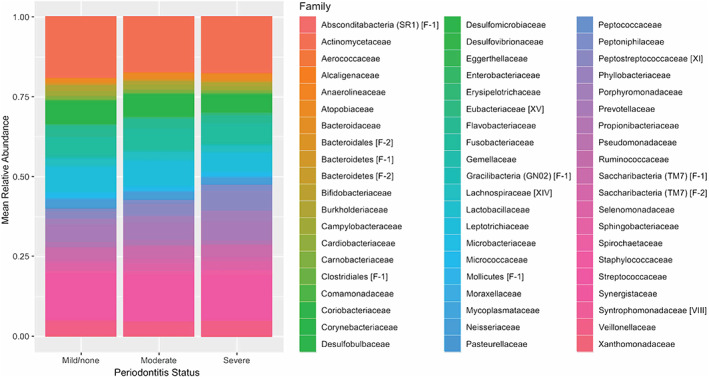
Phyloseq taxa distribution family‐level plot by periodontitis status.

**FIGURE 3:**
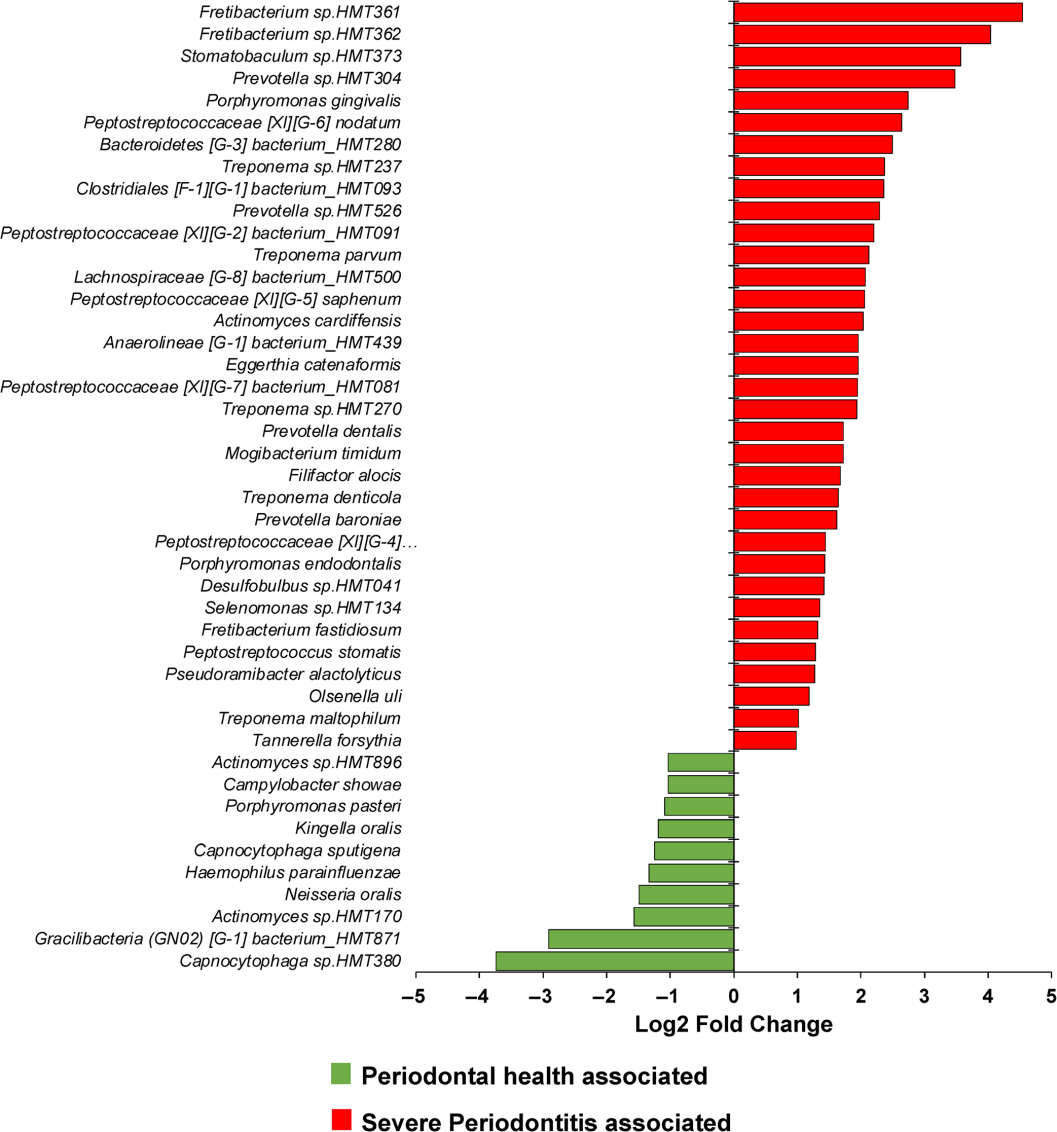
Results of DeSeq2 analysis comparing species abundance in samples from participants with mild or no periodontitis with samples from patients with severe periodontitis (*p*_adj_ < .01).

**FIGURE 4:**
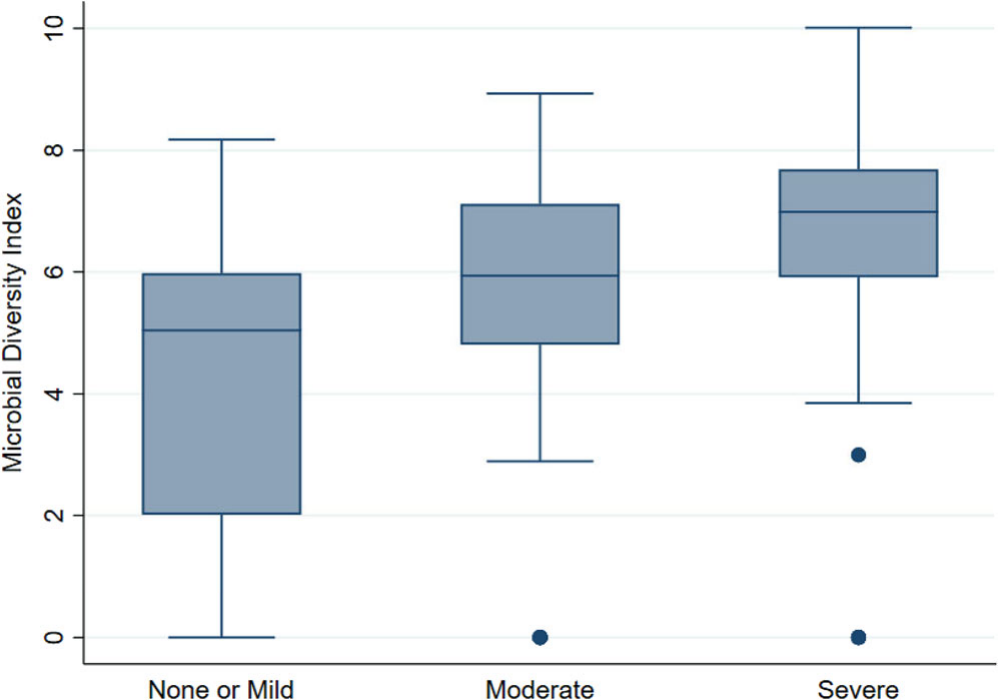
Boxplot depicting microbial diversity index (MDI) by Centers for Disease Control and Prevention and the American Academy of Periodontology (CDC/AAP) periodontitis status.

Using data obtained from the plaque 16S rRNA gene sequencing, the composition and abundance distributions of microbial taxa based on periodontitis status were obtained. Figure 2 shows the family‐level taxa distribution by periodontitis status. Subtle shifts across the relative abundance of various families are observed on moving from periodontal health to moderate periodontitis to severe periodontitis. DESeq2 was used to identify significant changes in abundance at the species level when severe periodontitis samples were compared to mild or no periodontitis samples (Figure 3). Significantly higher abundance (log_2_ fold‐change >1.2; *p*_adj_ <.01) of 34 species was identified in severe periodontitis samples, including typical red complex pathogens. Ten species exhibited significantly lower abundance in samples from severe periodontitis. These differentially abundant species were used to generate an MDI, corresponding to the log of (total abundance in organisms increased in severe periodontitis) over (total abundance of organisms decreased in severe periodontitis) (see Section 2). The boxplot in Figure 4 shows the derived MDI by periodontitis status. MDI increases across periodontitis categories, indicating a gain of taxa abundance associated with disease severity, while taxa with opposite association show decreased abundance.

Multiple linear regression analysis results are reported in Table 2. A unit increase in MDI equated to −0.88% (95% confidence interval [CI]: −1.49 to −0.28; *p* < .01) difference in % predicted FEV_1_ in the crude model. In the fully adjusted model (Model 4), this decreased to −0.71% (95% CI: 0.06%–1.35%; *p* = .03) difference in the % predicted FEV_1_. Confounding variables that were significantly associated with the % predicted FEV_1_ in the fully adjusted model also included smoking (*p* < .001), hypertension (*p* = .02) and education years (*p* < .01).

**TABLE 2:** Multiple linear regression investigating independent variables and their association with % predicted FEV_1_ (*n* = 507).

	Crude model, β‐coefficient (95% CI)	Model 1, β‐coefficient (95% CI)	Model 2, β‐coefficient (95% CI)	Model 3, β‐coefficient (95% CI)	Model 4, β‐coefficient (95% CI)
MDI (per unit increase)	**−0.88 (−1.49, −0.28)**	**−0.72 (−1.38, −0.06)**	**−0.79 (−1.44, −0.14)**	**−0.71 (−1.36, −0.06)**	**−0.71 (−1.35, −0.06)**
Mean PPD (per mm increase)		−1.84 (−4.83, 1.14)	−0.18 (−3.21, 2.85)	0.01 (−3.04, 3.05)	0.35 (−2.68, 3.39)
Toothbrushing frequency (ref. ≥two times/day)		−0.41 (−7.78, 6.96)	0.38 (−6.84, 7.60)	−0.50 (−7.72, 6.72)	−1.38 (−8.58, 5.82)
Age (per year increase)			−0.45 (−0.94, 0.04)	−0.43 (−0.92, 0.06)	−0.44 (−0.92, 0.05)
Waist circumference (per cm increase)			−0.04 (−0.07, 0.00)	−0.04 (−0.07, 0.00)	−0.03 (−0.07, 0.00)
Smoking status (ref. never smoker)					
Former smoker			**−6.17 (−9.42, −2.91)**	**−5.99 (−9.24, −2.74)**	**−5.86 (−9.10, −2.62)**
Current smoker			**−8.96 (−13.53, −4.39)**	**−8.74 (−13.28, −4.19)**	**−8.15 (−12.69, −3.60)**
Diabetes (ref. no)				2.24 (−3.79, 8.27)	2.63 (−3.46, 8.73)
Hypertension (ref. no)				**−3.69 (−6.92, −0.45)**	**−3.77 (−6.99, −0.54)**
ACVD (ref. no)				−4.08 (−8.71, 0.55)	−3.57 (−8.18, 1.04)
Material conditions (ref. Med/high)					−1.00 (−4.33, 2.32)
Education year (per year increase)					**0.68 (0.20, 1.16)**

*Note*: Model 1, adjusted for mean probing pocket depth and toothbrushing frequency; Model 2, Model 1 + adjusted for age, waist circumference, and smoking; Model 3, Model 2 + adjustment for history of diabetes, hypertension, and ACVD; Model 4, Model 3 + adjustment for material conditions, education years; Values in bold signify statistical significance *p* < .05.

Abbreviations: ACVD, atherosclerotic cardiovascular disease; CI, confidence interval; MDI, microbial diversity index; PPD, probing pocket depth; ref, reference category.

## 4 DISCUSSION

### 4.1 Main finding

The main finding of this cross‐sectional study was that subgingival microbial diversity, measured using the MDI, was significantly associated with reduced respiratory function as measured by the % predicted FEV_1_ in a group of middle‐aged men from Northern Ireland. For unit increase in MDI, there was a 0.71% loss (95% CI: 0.06%–1.35%; *p* = .03) in the % predicted FEV_1_ in the fully adjusted model. This finding may suggest that periodontitis‐associated microbial diversity may represent a putative risk indicator for respiratory decline.

### 4.2 Interpretation of results

Our initial hypothesis was that subgingival microbial changes associated with periodontitis may impact the lung microbiome, which may then result in reduced respiratory function. A recent review postulates that most common forms of periodontitis are associated with an increased bacterial diversity compared with periodontal health (Scannapieco & Dongari‐Bagtzoglou, 2021). High‐throughput sequencing studies have also reported that shifts in community structure from health to periodontitis resemble ecological succession, with emergence of newly dominant taxa in periodontitis without replacement of primary health‐associated species (Abusleme et al., 2013; Griffen et al., 2012). In this study, we operationalized the use of an MDI based on the clinical status of a participant's periodontal status. This is a ratio of more abundant taxa versus less abundant taxa relative to the periodontitis diagnosis, selected utilizing the DESeq2 package (Love et al., 2014). A one unit increase in MDI was associated with a decrease of 0.71% in respiratory function. While this may seem modest, it should be borne in mind that the range of the MDI was from 0 to 10. Additionally, variables such as years in full‐time education (per year increase) equated to a comparable magnitude of 0.68% increase in lung function (Table 2).

Despite our findings, one of the main limitations of the current study is the lack of a lung microbiome sample to analyse with respect to the oral sample. Aspiration of periodontal pathobionts themselves, aspiration of respiratory pathogens that have colonized periodontal pockets, aspiration of periodontal microbial components or their by‐products or some form of pathogenic interaction between periodontal microbes and lung microbes, all represent plausible pathways for the observed association (Scannapieco, 1999). As we have only studied the outcome with respect to respiratory decline, no assumptions can be made with respect to the composition of the lung microbiome. Sampling of the lung microbiome, however, is not without its challenges (O'Dwyer et al., 2016). It is impractical to directly obtain lung tissue for in situ microbiome analysis unless surgically justified, such as lung transplantation or tumour resection. Indirect methods such as bronchoalveolar lavage (BAL) carried out by bronchoscopy are still invasive, time consuming and costly, and therefore not practical in a population‐based cohort study. In a small study involving 29 participants who underwent BAL, Segal and co‐workers found increased community abundance of *Prevotella* and *Veillonella* (common anaerobic oral commensals) associated with higher levels of lymphocyte and neutrophil inflammation in the lung (Segal et al., 2013). Less invasive techniques, such as collecting a sputum sample, are also not ideal when dealing with a cohort that has a predominantly ‘healthy’ respiratory function. In the current study, only 10 (2%) men had a % predicted FEV_1_ of less than 50% indicating severe airflow limitation, demonstrating that the cohort did not have significant levels of respiratory disease. The contamination of sputum samples with oral saliva also represents an additional challenge when independence of samples would be required. Unrelated to investigating the oral microbiome, a recent small study (*n* = 48) was also able to demonstrate that in a group of healthy participants the composition of the lung microbiome analysed from sputum samples was independently associated with respiratory function (Lee et al., 2019).

### 4.3 Respiratory diseases and respiratory function

While each of the various types of respiratory diseases presents with distinct phenotypes and pathologies, a common feature of many respiratory diseases is a decline in respiratory function. In this study, we utilized the % predicted FEV_1_ as the main outcome variable of interest, as it factors in age, gender, height and race (Quanjer, 1983). FEV_1_ is the most widely used and quoted lung function test in clinical practice. It is easily measured and has very good reproducibility (Kerstjens et al., 1997). There is strong epidemiological evidence to indicate that reduced expiratory volume in 1 s (FEV_1_) is a marker of cardiovascular mortality, independent of age, gender and smoking history (Sin et al., 2005). Therefore, the implications of subgingival dysbiosis being a significant variable that is independently associated with reduced respiratory function are important. From a therapeutic point, if a causal relationship exists, there may be a role for therapies targeting oral microbiome changes including oral hygiene, periodontal therapy, denture hygiene, diet advice and so on. Obviously, further studies are required before arriving at this assertation.

### 4.4 Strengths of the study

The strengths of this study include the homogeneity of the sample, namely White Western‐European men. Ethnicity has previously been shown to have potential impacts on the human microbiome composition (Brooks et al., 2018), on periodontitis status (Delgado‐Angulo et al., 2016) and on respiratory disease featuring respiratory decline such as COPD (Behrendt, 2005). There was also a limited age range of 58–72 years, meaning similar time frames for potential life‐long exposure to other recognized respiratory risk factors such as indoor and outdoor air pollution, occupational hazards and infections (Mannino & Buist, 2007). Owing to the design of the PRIME study with its main aim to investigate the risks factors for ACVD, we were also able to make use of a range of other data on potential confounding factors in the current study (such as smoking status, anthropometric measurements, co‐morbidity status and socio‐economic variables). A further strength relates to the highly standardized method in obtaining comprehensive lung function parameters using standard spirometry. The use of the % predicted FEV_1_ as the primary outcome variable as opposed to the non‐standardized FEV_1_ measure also adds further robustness to the analysis. To our knowledge, the present study is the first investigating the association between microbial diversity and the % predicted FEV_1_.

### 4.5 Limitations of the study

There are several limitations to this study. First, this study was conducted in a group of White Western‐European men, which limits the generalizability of study findings. Second, the study is cross‐sectional and, as such, it is not possible to determine whether subgingival microbial diversity contributed to reduced respiratory function or was a consequence of it. Third, the current study (*n* = 507) is a sub‐study of the main PRIME study (*n* = 2748). This has the potential to introduce selection bias, affecting how representative the men were of the original cohort. Given the age range of this group of men, a number of them had died, and others might not have been able to re‐attend because of illness. A supplementary analysis (Table S1) using data gathered during the original recruitment (1991–1994) revealed that the participants included in the current study were marginally younger, had a greater proportion of never/former smokers, were from a better socio‐economic background and had marginally better FEV_1_ values. Another form of selection bias relates to the restricted age range of men in the current study (58–72 years). The higher prevalence of comorbidities in comparison with younger men means that they will be unrepresentative of the general population. Fourth, there could also be measurement bias. Training and calibration of the dental hygienists involved in the study attempted to maintain reproducibility, but we acknowledge that error may still exist in both the periodontal examinations and subgingival plaque sampling. Fifth, a non‐standardized approach to plaque sampling was used. Plaque sampling based on deepest accessible diseased sites may have an effect of biasing the study towards those with periodontitis. Owing to several characteristics, such as oxygen availability, the microbiome in deeper pockets may differ greatly from that in shallow pockets. However, as discussed previously, for some individuals exhibiting a healthy periodontium with shallow pocket depths, a high degree of MDI was still observed (Figure 4). Furthermore, pooling of samples on a patient basis for NGS analysis may also have had an effect on the observed microbial results. Sixth, the novel use of a subgingival MDI in the context of this study requires further validation in future studies. Finally, in common with all observational studies, the possibility of residual confounding or failure to account for other potential confounders exists. Furthermore, it is acknowledged that in our statistical analysis, the difference in regression coefficients from the simple to the adjusted models could be evidence of dependency between the variables through mediation and/or confounding.

## 5 CONCLUSIONS

In conclusion, subgingival microbial diversity was found to be associated with reduced respiratory function in a group of 58–72‐year‐old men from Northern Ireland. This relationship was independent of known confounders and, as such, could reflect the possibility that subgingival microbial diversity associated with periodontitis may be an aetiological factor for respiratory decline. Alternatively, there may be shared biological pathways between subgingival microbial diversity and respiratory decline. Further studies should be aimed at specifically addressing the issue of causality and whether periodontitis‐associated changes in the oral microbiome directly negatively impacts on the lung microbiome. Studies are also required to investigate whether prevention or treatment of periodontitis might have a beneficial impact on respiratory function.

## AUTHOR CONTRIBUTIONS

Gerard J. Linden and Frank Kee devised the study, developed the main conceptual ideas and oversaw the PRIME Belfast study. Gerard J. Linden oversaw all periodontal examinations. Kathy M. Cullen validated all respiratory examinations, with Dermot Linden providing respiratory analysis. Mary McClory, Ikhlas El Karim and Fionnuala T. Lundy were responsible for processing plaque samples and carrying out next‐generation sequencing. Gary Moran provided bioinformatics expertise. Lewis Winning, Gary Moran and Christopher C. Patterson undertook the statistical analysis. Lewis Winning drafted the initial version of the manuscript. All authors reviewed and approved the final version of the manuscript.

## FUNDING INFORMATION

This study was supported by grants from the British Heart Foundation (PG/14/9/30632), the Northern Ireland Research and Development Fund (RRG 5.22) and the Heart Trust Fund (Royal Victoria Hospital, Belfast). Next‐generation sequencing was supported by a grant from the British Endodontic Society.

## CONFLICT OF INTEREST STATEMENT

The authors declare no conflict of interest.

## Supporting information


**Table S1.** Comparison of baseline characteristics at original recruitment into the PRIME study (1991–1994) of participants included in current analysis versus those not included.
**Table S2.** Comparison of characteristics at re‐screening (2001–2003) of dentate participants (*n* = 1400) who had a periodontal examination and were included in the current analysis versus those not included.

## Data Availability

The data that support the findings of this study are available from the corresponding author upon reasonable request.
