# Supplementary material for: Subgingival microbial diversity and respiratory decline: A cross‐sectional study
Source: J Clin Periodontol. 2023 Apr 13;50(7):921–31. doi: 10.1111/jcpe.13819 (PMC10947346; doi:10.1111/jcpe.13819)
Supplement: Supplementary file 1 — Table S1. Comparison of baseline characteristics at original recruitment into the PRIME study (1991–1994) of participants included in current analysis versus those not included. Table S2. Comparison of characteristics at re‐screening (2001–2003) of dentate participants (n = 1400) who had a periodontal examination and were included in the current analysis versus those not included. [file JCPE-50-921-s001.docx]

Supplementary Information

**Supplemental table S1.** Comparison of baseline characteristics at original recruitment into PRIME study (1991-1994) of participants included in current analysis versus those not included.

|  | Current study sample (n=507) | Remainder of original study sample  (n=2,241) | *p* |
| --- | --- | --- | --- |
| Age, years, mean (SD) | 54.2 (2.8) | 54.9 (2.9) | **<0.01** |
| BMI, kg/m^2^, mean (SD) | 26.3 (3.2) | 26.2 (3.4) | 0.61 |
| Smoking, *n* (%)  Never  Former  Current | 198 (39.4%)  194 (38.6%)  110 (21.9%) | 691 (31.7%)  750 (34.3%)  738 (33.9%) | **<0.01** |
| Diabetes, *n* (%) | 63 (6.7%) | 50 (5.6%) | 0.40 |
| Materials Conditions, *n* (%)  Low  Medium  High | 153 (30.2%)  127 (25.1%)  226 (44.7%) | 900 (41.3%)  447 (20.5%)  831 (38.1%) | **<0.01** |
| FEV_1_, mean % (SD) | 3.2 (0.7) | 3.1 (0.7) | **<0.01** |
| FVC, mean % (SD) | 4.2 (0.8) | 4.1 (0.8) | 0.06 |

**Supplemental table S2.** Comparison of characteristics at rescreening (2001-2003) of dentate participants (n=1400) that had a periodontal examination and were included in current analysis versus those not included.

|  | Had plaque sampling (n=507) | Did not have plaque sampling (n=893) | *p* |
| --- | --- | --- | --- |
| Age, years, mean (SD) | 63.6 (3.1) | 63.8 (2.9) | 0.16 |
| BMI, kg/m^2^, mean (SD) | 27.6 (3.6) | 27.5 (3.6) | 0.60 |
| Smoking, *n* (%)  Never  Former  Current | 204 (40.2%)  222 (43.8%)  81 (16.0%) | 351 (39.3%)  388 (43.4%)  154 (17.2%) | 0.82 |
| Diabetes, *n* (%) | 34 (6.7%) | 50 (5.6%) | 0.40 |
| Materials Conditions, *n* (%)  Low  Medium  High | 153 (30.2%)  127 (25.1%)  226 (44.7%) | 305 (34.2%)  198 (22.2%)  390 (43.7%) | 0.25 |
| Moderate / Severe Periodontitis, *n* (%) | 203 (40.0%) | 388 (43.4%) | 0.21 |
| Number of teeth, mean (SD) | 19.4 (5.9) | 19.4 (6.2) | 0.99 |
| % predicted FEV_1_, mean % (SD) | 92.5 (17.5) | 90.7 (17.7) | 0.07 |
